# Supplementary figures and images for: From heterogeneous healthcare data to disease-specific biomarker networks: A hierarchical Bayesian network approach
Source: PLoS Comput Biol. 2021 Feb 12;17(2):e1008735. doi: 10.1371/journal.pcbi.1008735 (PMC7906470; doi:10.1371/journal.pcbi.1008735)

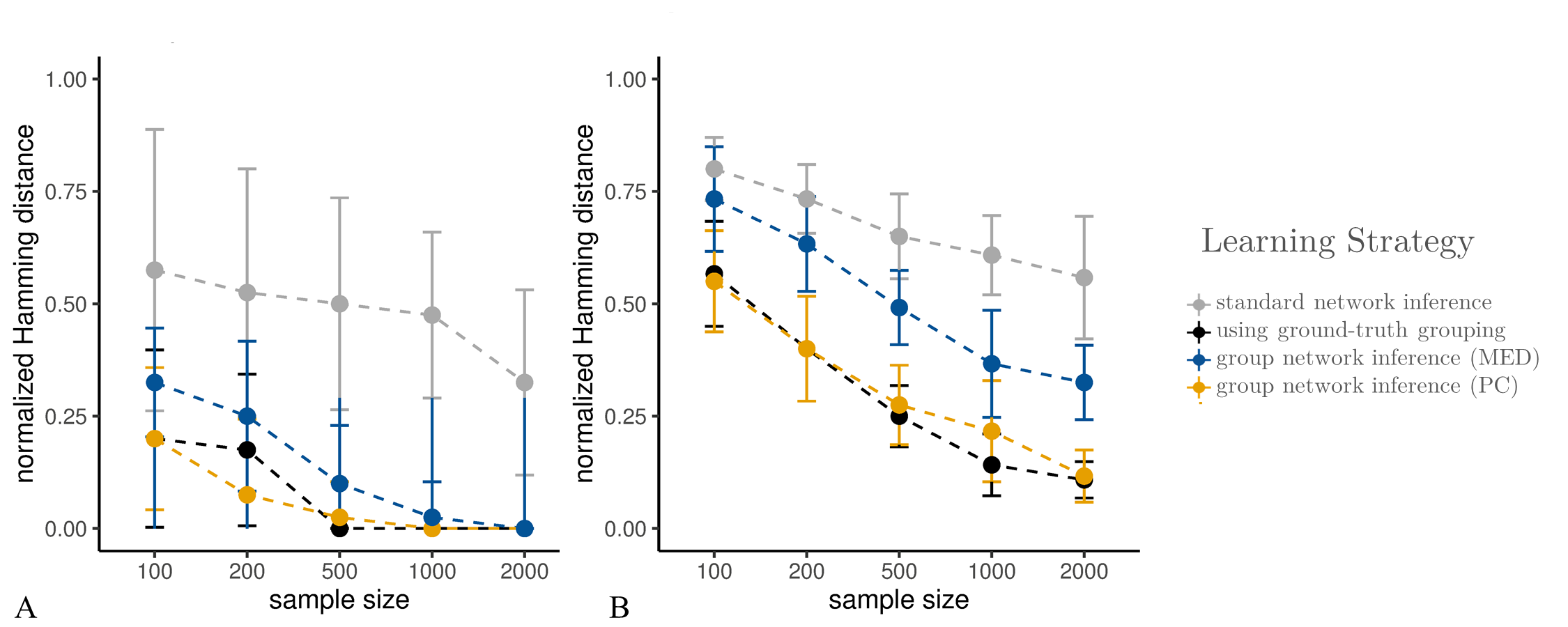

Supplement: S1 Fig — Results of the reconstruction of group networks for varying sample sizes. A Group networks with 5 nodes.B Group networks with 20 nodes. On the basis of these simulations, we decided to run the remaining simulations with group networks of size 20 and a medium sample size of 500. (TIF) [file pcbi.1008735.s001.tif]

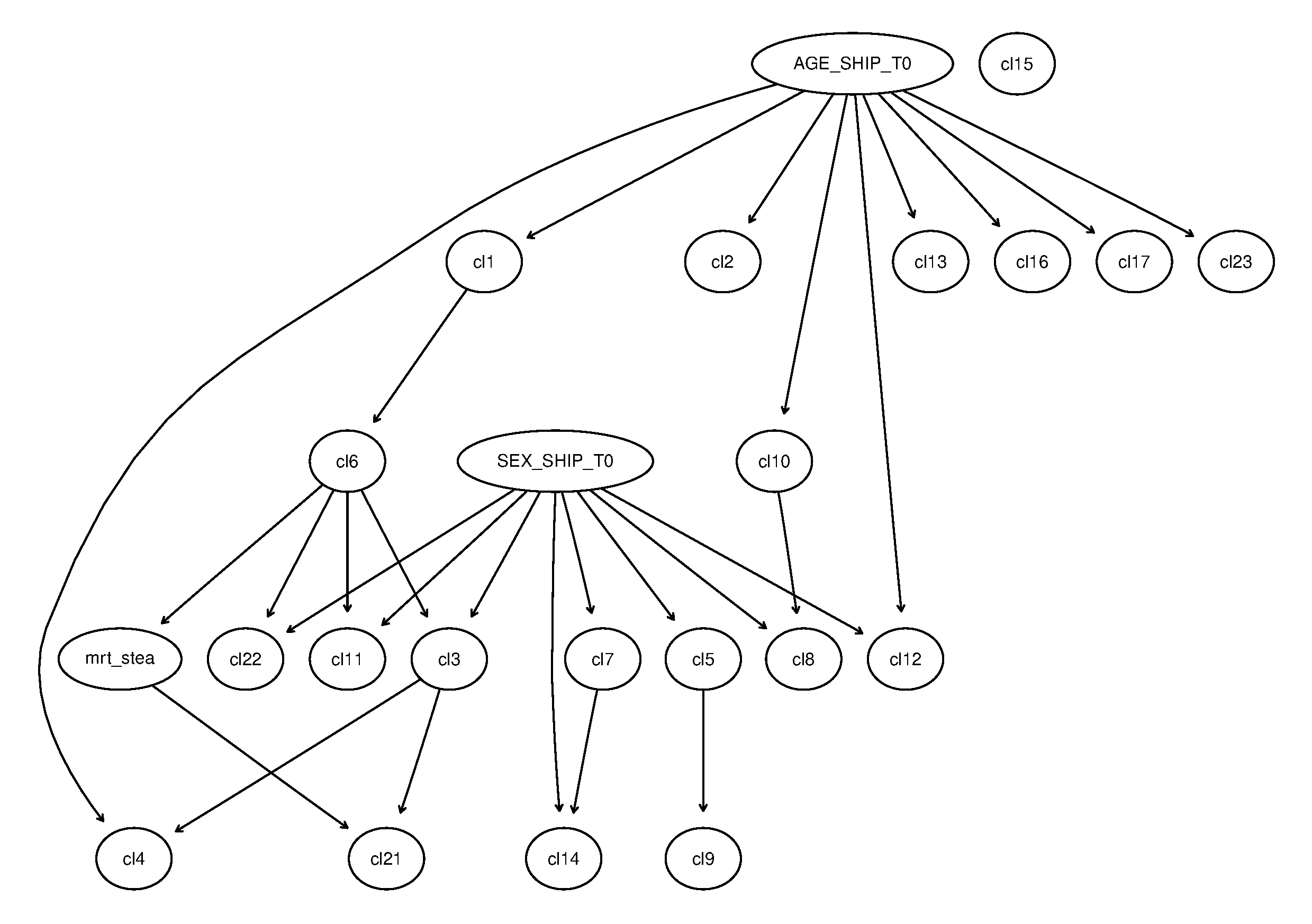

Supplement: S2 Fig — Group Bayesian network with target variable steatosis. (TIF) [file pcbi.1008735.s002.tif]

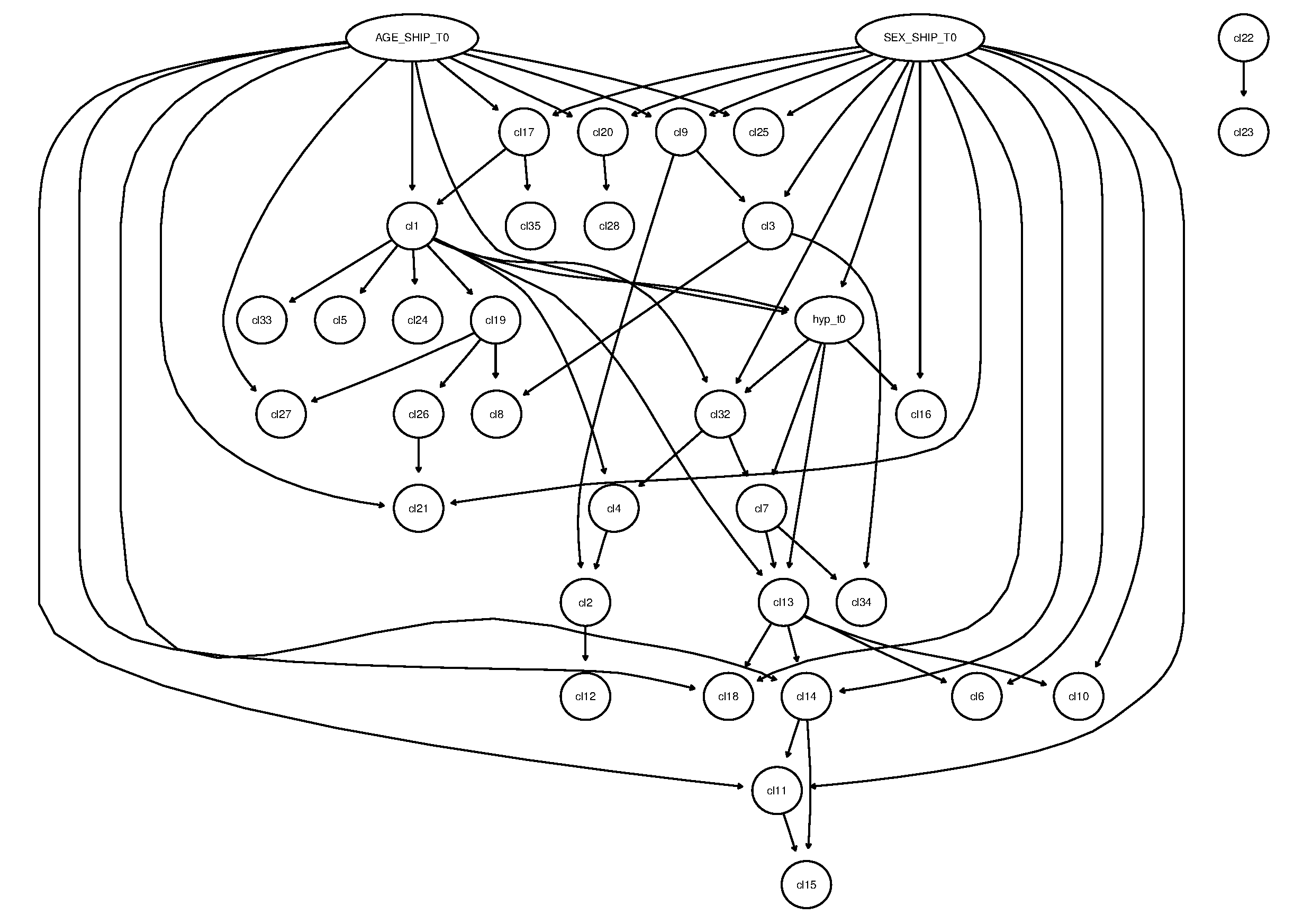

Supplement: S3 Fig — Group Bayesian network with target variable hypertension. (TIF) [file pcbi.1008735.s003.tif]

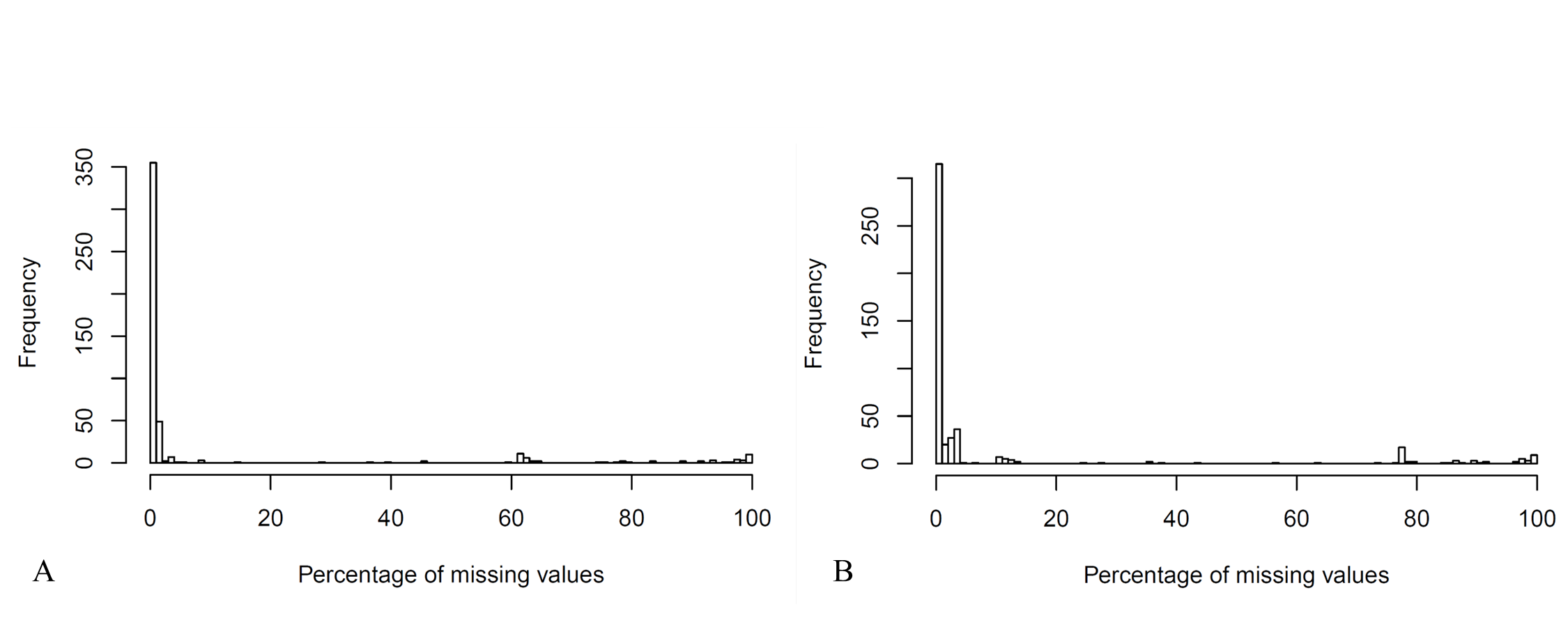

Supplement: S4 Fig — Histograms of missing values in % per variable A for the subset of participants included in steatosis model B for the subset of participants included in hypertension model. (TIF) [file pcbi.1008735.s004.tif]
