# Supplementary material for: From heterogeneous healthcare data to disease-specific biomarker networks: A hierarchical Bayesian network approach
Source: PLoS Comput Biol. 2021 Feb 12;17(2):e1008735. doi: 10.1371/journal.pcbi.1008735 (PMC7906470; doi:10.1371/journal.pcbi.1008735)
Supplement: Attachment — Submitted filename: response.pdf [file pcbi.1008735.s007.pdf]

Response to review of the submitted article PCOMPBIOL-D-20-01495

Dear Alison Marsden, dear Florian Markowetz,

we would like to thank you and the two reviewers for the evaluation of our manuscript. We are very pleased with the positive feedback and have addressed the constructive comments by making appropriate changes to the manuscript. Before giving a point-by-point response to all comments, the main changes are shortly summarized in the following.

- We complemented the information on the preprocessing of the data and the analysis of the simulated data.
- We added a table for comparison of different hypertension models

Reviewer 1 has pointed out that data for the NAFLD and hypertension studies is not provided with the paper or supplemental material. These dataset derive from the Study of Health in Pomerania (SHIP) and contain potentially identifying and sensitive information on the study participants, we not allowed to publicly discluse these data sets. However, data access can be requested for research purposes from the community medicine data access committee of University Medicine Greifswald, details on the application procedure and the online application form can be found at http://fvcm.med.uni-greifswald.de
In closing, we again thank the associate editor and the reviewers for the possibility to resubmit, and hope that in its revised form, the paper will be suitable for publication in PLOS Computational Biology.

Yours sincerely,
Lars Kaderali

Response to Reviewer 1

- *The paper proposes an automatic algorithm that hierarchically refines the structure of a Bayesian network to detect groups of homogeneous features and to learn their conditional relation. After an initial presentation of the algorithm, the authors compare the performance of their approach on a synthetic dataset generated from a parametric family of networks, and assess the ability of the proposed approach to recover the original Bayesian network topology and parameters. Their approach is then compared with other strategies to handle groups in Bayesian networks and by aggregation strategies using medoids and first principal components. The proposed approach is tested on a toy model which is used to determine factor distinguishing wines produced from two different types of soils. This is followed by an analysis of a large collection of electronic health records, focusing on two conditions whose early diagnosis is critical for positive long term outcomes, i.e., non-alcoholic fatty liver disease and systemic hypertension. Refined group Bayesian networks show superior performance than commonly adopted clinical indices, logistic regression and Bayesian networks with different group handling strategy.*

  The paper is interesting and well written. Publication is recommended, I have only a few minor comments for the authors to address.

We thank Reviewer 1 for the positive evaluation of our manuscript and for the helpful comments. We took advantage of these when preparing the current revision. The comments of Reviewer 1 are adressed below.

THINGS THAT ARE NOT CLEAR OR SHOULD BE EXPLAINED FURTHER

- *pag. 5 section Evaluating simulated data. While the difference between approaches to aggregate groups (i.e., medoids and first principal components) is clear, the exact meaning of the other methods such as 'network-based' and 'using group data' in Figure 3 is not clear. Is 'network-based' an approach where group information is disregarded and group Bayesian network an approach where the group separation is initially a-priori enforced and left unaltered? The authors should further explain the nature of the approaches they are comparing against, adding appropriate citations from the literature, if appropriate.*

  Thank you for your pointing out, that the descriptions of the simulations were not precise enough. We renamed the learning approaches and added further information to the description of Figure 3, to lines

121–141, and to the respective method section (lines 468–478).
In summary, the 'standard network inference' approach (former 'network-based') describes the learning of a large, detailed BN from the sampled data directly. The grouping and the group structure are then only identified post-hoc from this network. On the contrary, our proposed algorithm ('group network inference') includes a step of data-based clustering and group aggregation prior to network inference. To avoid confusion, 'Using group data' was renamed to 'using ground-truth grouping'. Here, the original group variables are used to infer a group network structure directly from layer 0. This step was added, as it is often impossible to learn the completely correct network from data, even when they were sampled from the original variables. The comparison to a Hamming distance of 0 could thus be misleading.

- *In my opinion, the policy of the journal where the result section precedes the method section is suboptimal for this paper. Many questions that arise in terms of precisely defining how the numerical tests are performed on both synthetic and real datasets find answers in the method section. I therefore think the paper would benefit from switching the result and method sections*

  We agree with Reviewer 1 that methodological details remain open while reading the results. However, we wanted to ensure that the main story is readable even without the very technical background. We consulted about this question also with the editorial office, and after their feedback decided to sticked to the journal guidelines - we hope the reviewer will find this decision acceptable.

- *I was wondering if the authors could add some more detail on how exactly the predictions from the Bayesian network model were obtained in Table 1. Were all other variables assigned as evidence and the most likely value of the variable steatosis inferred using a max-product algorithm? Or just variables belonging to the Markov blanket of the variable steatosis were used, independent on observations on other variables being missing?*

  Predictions from Bayesian network models were obtained by taking all nodes except for the target as evidence. The respective posterior probability was estimated using likelihood weighting. We added this information to the description of Table 1. We also added a note to the method section (line 365).

- *It is not clear why a table like Table 1 is not provided for the application on systemic hypertension.*

  Please see the detailed answer to Question 4 of Reviewer 2.

- *The authors should report the processing times required for the two real dataset analyzed in the papers, i.e., 2311-407 and 4403-328 participant-features for the NAFLD and hypertension datasets, respectively. Specifically, they should report the time required to perform the initial hierarchical clustering, structure learning with group refinement, parameter learning and prediction.*

  We thank Reviewer 1 for pointing out that processing times were missing. We now placed the subsection *Computations and code availability* at the end of the method section (lines 532–535) and added information on processing times for the NAFLD and Hypertension model. We state separate times for the initial hierarchical clustering, the learning of the initial group network, as well as the mean time spent on one refinement iteration (including regrouping, structure learning, and parameter estimation for all neighboured models). However, it must be noted, that the processing times are highly dependent on the complexity of the chosen structure learning algorithm, the number of groups and the number of neighboured models.

SYNTAX, ETC.: TITLE, ABSTRACT, REFERENCES

- *Title and abstract appear to be appropriate*
  Please review reference 15.

  Reference 15 was discarded. We apologize for the superfluous reference.

Response to Reviewer 2.

We thank Reviewer 2 for the positive evaluation of our paper and for the helpful remarks. Below we address the comments point-by-point.

Major Comments

- *The AUROC for the unrefined detailed Bayesian network was significantly lower for hypertension than for NAFLD. Why are these thought to be so different?*

  In general, the prediction from a detailed network depends largely on the connection of the target to the rest of the network. The final group network for hypertension suggests that the risk of hypertension is dependent on a larger set of predictors, with lower individual influence. Presumably, these connections were not properly learned in a generative approach as many additional arcs would be necessary, that do not increase the overall score a lot. In contrast, the strong dependence of NAFLD on the liver function tests allowed for better propagation in a detailed network, resulting in a higher AUROC. We added an according note to lines 292–294.

- *For the SHIP Trend data used, it would be useful if the authors provided a little more detail on the input variables and indicate the total number of variables. Were these the same for both the hypertension and NAFLD analyses?*

  The original set of features was the same in both examples. However, preprocessing steps included the removement of specific context-related variables and, therefore, differed for both models. We added further information on this step to the respective method section (lines 500–524). The total number of features and participants for each model are now also given in Table 2.

- *For the SHIP Trend data used, how common were missing variables in the data set used for the analyses? The manuscript indicates that for the both clinical analyses variables with greater than 20 missing data were excluded. Do the authors have any estimate of the effect that missing variables had on their results?*

  The threshold of 20% was chosen with the intention to remove those features that were measured for specific patient subgroups only and thus are missing not at random (e.g., hormone measurements, differential haematology). We added histograms showing the percentages of missing values (S6 Fig.) to the supplement, that support the decision on the threshold. We also added further information on the numbers of removed features to the manuscript (lines 500–524).

- *The results for the NAFLD analyses were compared with NAFLD clinical risk prediction models. Why wasn't the same done with the hypertension analyses? A number of such models have been reported.*

  To our best knowledge, established clinical risk scores (e.g., as reported in [1] or [2]) aim at predicting future hypertension based on current blood pressure and further covariates. This approach differs significantly from ours, as we identify factors associated with currently present hypertension. A direct comparison with these clinical scores is therefore not possible. To nonetheless provide some more details on model performance, we added a table (now Table 3) for the hypertension model that includes prediction scores from three different Bayesian network models and we compared our approach to a regularized logistic regression.

[1] Sun D, Liu J, Xiao L, Liu Y, Wang Z, et al. Recent development of risk-prediction models for incident hypertension: An updated systematic review. PLOS ONE 2017; 12(10): e0187240. doi:10.1371/journal.pone.0187240

[2] Echouffo-Tcheugui JB, Batty GD, Kivimäki M, Kengne AP. Risk models to predict hypertension: a systematic review. PLOS ONE 2013; 8(7):e67370. doi:10.1371/journal.pone.0067370.

Minor Comments

- *The clinical standard is to diagnose hypertension when the blood pressure is elevated on repeated measurements rather than a single measurement. Was that the case for the subset of patients diagnosed as being hypertensive based on blood pressure readings?*

  In SHIP-Trend, three repeated BP measurements were conducted. The first one was discarded to reduce effects like white coat hypertension. The average over the latter two measurements was used for diagnosis. We added this information to the paper (lines 513–514).

- *AUROC and AUPRC are only defined in a figure caption, not in the text.*

  We thank Reviewer 2 for pointingt this out. The abbreviations are introduced in the revised version of the manuscript (lines 238–243, and in the related method subsection lines 491–494).

- *In Figure 5, BIA is not defined.*

  The abbreviation was removed and the full term is used, instead.
